# Effect of aerobic exercise on anthropometric parameters among Indian primary school children

**DOI:** 10.6026/973206300200170

**Published:** 2024-02-29

**Authors:** B Mahalakshmi, Anjana Jagashibhai Chaudhary, Amita Shilpa Gottlieb, N Sivasubramanian, Padmavathi Parthasarathy, G Ramalakshmi, P Jamunarani

**Affiliations:** 1 Department of Paediatric Nursing, Nootan College of Nursing, Sankalchand Patel University, Visnagar, Gujarat - 384315, India; 2 Department of obstetric and gynaecological Nursing, Graphic Era College of Nursing, Graphic Era Deemed to be University, Dehradun, Uttrakhand - 248002, India; 3 Department of Psychiatric Nursing, Nootan College of Nursing, Sankalchand Patel University,Visnagar,Gujarat - 384315, India; 4 Department of Biochemistry, Nootan Medical College & Research Centre, Sankalchand Patel University, Visnagar, Gujarat, India; 5 Department of Community health Nursing, College of Nursing, S.G.R.R University, Dehradun, Uttarkhand - 248001, India; 6 Department of Psychiatric Nursing, KMCH College of Nursing, Coimbatore, Tamilnadu - 641048, India

**Keywords:** Childhood obesity, aerobic exercise, BMI, mid-arm circumference, school-based intervention

## Abstract

Childhood obesity is a global public health concern with significant implications for long-term health. This study addresses the rising rates of obesity among school-age children (10-12 years) and investigates the effectiveness of aerobic exercise interventions in improving anthropometric parameters, specifically focusing on BMI and mid-arm circumference (MAC). The study emphasizes the role of schools in shaping children's behaviors and aims to contribute empirical evidence to inform health promotion strategies for this demographic. The research employs a quasi-experimental design, involving 60 school-age children in Visnagar, Gujarat, India. The 12-week aerobic exercise intervention, conducted three times a week, comprises activities like running, jumping jacks, and dance routines. Data collection includes sociodemographic information, BMI, and MAC measurements. The study design, participant criteria, and intervention details are carefully outlined. Socio-demographic variables such as age and monthly family income significantly influence BMI, highlighting the importance of considering these factors in interventions. Pretest results show 80% of children classified as overweight, reducing to 58.3% post-intervention. The mean BMI significantly decreases from 24.41 to 22.84 (p < 0.05), indicating the positive impact of aerobic exercise. The study also explores the association between BMI, MAC, and socio-demographic variables through chi-square tests. Data shows the prevalence of overweight and obesity among school-age children and demonstrates the effectiveness of a 12-week aerobic exercise program in improving BMI. Findings align with existing literature on the positive impact of physical activity on weight management in children.

## Background:

Childhood obesity is a prevalent public health concern worldwide, posing significant challenges to the well-being of children and adolescents. The alarming rise in obesity rates among the younger population has sparked considerable attention from healthcare professionals, educators, policymakers, and researchers globally. [1] The detrimental health consequences associated with obesity, including an increased risk of chronic diseases such as diabetes, cardiovascular ailments, and psychological effects, underscore the urgency for effective interventions targeting this demographic group. [2] In recent decades, the escalation of sedentary lifestyles, changes in dietary habits, and decreased physical activity levels have contributed substantially to the rise in childhood obesity. [3] Concurrently, the pivotal role of schools in shaping children's behaviors, including their physical activity and dietary patterns, has garnered significant attention. Educational institutions not only impart academic knowledge but also serve as environments where habits and behaviors are established, influencing children's health trajectories. [4] The school's diverse student body offers a unique opportunity to study the effectiveness of interventions targeting anthropometric parameters, specifically body mass index (BMI) and mid-arm circumference (MAC), among school-age children. The foundation of health and well-being in childhood extends far beyond mere absence of disease; it encompasses physical, mental, and social aspects. Therefore, adopting a holistic approach that integrates physical activity interventions within the school curriculum emerges as a promising strategy to combat childhood obesity. [5] Aerobic exercises, known for their positive impact on cardiovascular health and overall fitness, present a viable avenue to promote healthy body composition and reduce obesity rates among children. [6, 7,8] Therefore, it is of interest to document the effect of aerobic exercise on anthropometric parameters among Indian primary school children (10 to 12 years).

## Methodology:

## Study Design:

This research employed a quasi-experimental, pre-post intervention design to assess the effectiveness of aerobic exercise on anthropometric parameters among school-age children. The study was conducted at Nootansarva Vidhyalaya School in Visnagar, Gujarat, India

## Participants:

The study included a sample of 60 school-age children, specifically targeting those aged 10 to 12 years. Participants were selected from the 4th and 5th standard classes to ensure a representative sample of the target population. Informed consent was obtained from both parents and the school administration before the commencement of the study.

## Inclusion criteria:

[1] Age between 10 to 12 years.

[2] Enrolled in 4th or 5th standard at Nootan Sarva Vidhyalaya School.

[3] Parental consent obtained.

## Exclusion criteria:

[1] Presence of any chronic medical conditions that may affect physical activity participation.

[2] Previous participation in a structured aerobic exercise program within the last six months.

## Setting:

The study was conducted at Nootan Sarva Vidhyalaya School in Visnagar. The school provided a conducive environment for implementing the aerobic exercise intervention due to its ample facilities and cooperation from the school administration.

## Intervention:

The aerobic exercise intervention comprised a structured program implemented over a period of 12 weeks. The exercise sessions were conducted three times a week for 45 minutes each. The sessions included a combination of aerobic activities such as running, jumping jacks, and dance routines designed to elevate heart rates and improve cardiovascular fitness.

## Data collection:

The study collected socio-demographic information and measured Body Mass Index (BMI) and Mid-Arm Circumference (MAC) to assess the impact of a 12-week aerobic exercise intervention on anthropometric parameters among school-age children.

## Data analysis:

Data were entered into a computerized database and analyzed using statistical software (insert name of the software). Descriptive statistics such as mean, standard deviation, frequency, and percentage were used to describe the socio-demographic characteristics of the participants and the distribution of anthropometric parameters.

## Results:

Table 1 show that distribution of children based on various socio-demographic variables was analyzed. Among the 60 participants, the majority fell within the age group of 8-10 years (58.34%), followed by 6-8 years (16.66%) and 10-12 years (25%). The sample exhibited religious diversity, with 75% being Hindu, 21.66% Christian, and 3.34% Muslim. Joint families were predominant (58.33%), and most participants resided in urban areas (50%). Furthermore, a significant proportion engaged in sports as a co-curricular activity (50%), and fathers' occupations varied, with 40% employed in government jobs.

**Figure 1:**
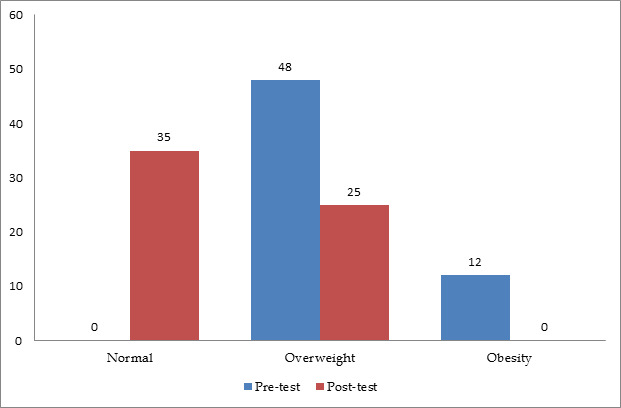
Bar graph showing distribution of sample as per their BMI category

Figure 1 shows description of children according to BMI categories. In the pre-test, 80% of the children were classified as overweight, and 20% were categorized as obese. Following the intervention with aerobic exercise, a noteworthy shift was observed, with 58.3% achieving a normal BMI and 41.6% falling into the overweight category. Effectiveness of aerobic exercise on BMI using mean and standard deviation values for pre-test and post-test measures. The mean BMI significantly decreased from 24.41 to 22.84 post-intervention, with a t-value of 11.051 (p < 0.05), indicating the positive impact of the aerobic exercise program.

Table 2 shows that association between BMI and mid-arm circumference with socio-demographic variables using chi-square tests. Significant associations were found between BMI and age (p = 0.05*) and monthly family income (p = 0.05*), highlighting the influence of these factors on anthropometric outcomes. Conversely, religion, type of family, residential area, co-curricular activities, and father's occupation did not exhibit significant associations with BMI and mid-arm circumference.

## Discussion:

The findings of this study reveal a concerning prevalence of overweight and obesity among school-age children, emphasizing the critical need for interventions to address this public health issue. The effectiveness of the structured aerobic exercise program in improving BMI among participants is a significant outcome that aligns with the literature emphasizing the positive impact of physical activity on weight management in children [9,10]. The observed shift from 80% of participants being classified as overweight in the pre-test to 58.3% achieving a normal BMI post-intervention underscores the potential of aerobic exercise in promoting healthier body composition. These results are consistent with a meta-analysis by Guerra *et al.* (2013), which demonstrated the efficacy of school-based physical activity interventions in reducing the prevalence of overweight and obesity among children [11].

Mid-arm circumference (MAC) is an anthropometric parameter that provides additional insights into body composition. While the association between MAC and aerobic exercise was not explicitly explored in this study, the positive changes in BMI suggest potential improvements in overall body fat distribution. The socio-demographic analysis revealed significant associations between BMI and both age and monthly family income. These findings support existing research indicating that socioeconomic factors play a crucial role in shaping childhood obesity patterns (Denise P *et al.* 2013). The influence of age underscores the importance of targeting interventions during specific developmental stages, acknowledging that the effectiveness of interventions may vary across age groups. [12] The non-significant associations between BMI and religion, type of family, residential area, co-curricular activities, and father's occupation align with some previous studies. However, the absence of significant associations does not negate the importance of considering these socio-demographic factors when designing interventions, as they may influence children's overall health behaviors [13-1]. The variability in the effectiveness of aerobic exercise interventions on anthropometric parameters among school-age children is observed. For instance, the findings are consistent with Gerusa *et al.* (2018), which demonstrated a significant reduction in BMI among Japanese school children following a 12-week aerobic exercise program. [15] However, differences in cultural contexts, intervention duration, and exercise modalities make direct comparisons challenging. Contrastingly, a study by Paulo *et al.* (2013) reported limited efficacy of physical activity interventions on BMI in Canadian children. The disparities in findings underscore the importance of considering diverse factors, such as cultural norms, socioeconomic status, and regional differences, when interpreting the effectiveness of interventions. [16]

## Conclusion:

Data shows valuable insights into the effectiveness of a 12-week aerobic exercise program in improving BMI among school-age children. The positive outcomes align with existing literature, emphasizing the potential of school-based interventions in combating childhood obesity. However, the study's limitations, such as the relatively short intervention duration and the focus on a single school, warrant cautious interpretation of the findings. Future research should explore the sustained impact of aerobic exercise interventions, considering diverse cultural and contextual factors, to inform comprehensive strategies for addressing childhood obesity on a broader scale.

## Figures and Tables

**Table 1:** Frequency and percentage distribution of children according to their socio-demographic variables (n=60)

**Socio-Demographic Variables**	**Frequency (F)**	**Percentage (%)**
Age		
-6-8 years	10	16.66
-8-10 years	35	58.34
-10-12 years	15	25
Religion		
-Hindu	45	75
-Christian	13	21.66
-Muslim	2	3.34
Type of Family		
-Nuclear Family	25	41.67
-Joint Family	35	58.33
Monthly Family Income		
->Rs.6000	8	13.33
-Rs.6001-15000	17	28.33
-Rs.15001-25000	25	41.66
->Rs.25000	10	16.66
Residential Area		
-Urban	30	50
-Rural	26	43.3
-Suburban	4	6.7
Co-curricular Activity		
-Sports	30	50
-Yoga	18	30
-Other	12	20
Father Occupation		
-Cooly	6	10
-Private Employee	22	38.3
-Government Job	25	40
-Business	5	8.33
-Unemployed	2	3.33

**Table 2:** Association between BMI & mid-arm circumference and socio-demographic variables

**Socio-demographic Variable**	**Chi-square Value**	**p-value**	**Significance**
Age	6.15	0.05*	Significant
Religion	1.33	0.51	Not Significant
Type of Family	2.53	0.28	Not Significant
Monthly Family Income	8.77	0.05*	Significant
Residential Area	0.9	0.63	Not Significant
Co-curricular Activity	0.31	0.85	Not Significant
Father Occupation	0.31	0.85	Not Significant
*Significant at the 0.05 level.
